# Multi-omics unveils seasonal remodeling and metabolic crosstalk between testis and abdominal fat body in a non-amplexus stream frog *Nanorana taihangnica* (Anura: Dicroglossidae)

**DOI:** 10.1186/s12864-026-13080-4

**Published:** 2026-06-22

**Authors:** Yimin Zhang, Ruinan Zhao, Xinyue Yang, Tonghang Wang, Yanjun Zhu, Yinmeng Hou, Zhuo Chen, Xiaohong Chen

**Affiliations:** 1 The Observation and Research Field Station of Taihang Mountain Forest Ecosystems of Henan Province, College of Life Sciences, Henan Normal University, No. 46 East Jianshe Road, Xinxiang, Henan 453007 China; 2 School of Basic Medical Sciences, Henan Medical University, No. 601 Jinsui Avenue, Xinxiang, Henan 453003 China

**Keywords:** Seasonal variation, Fat body, Testis, Transcriptomics, Metabolomics, *Nanorana taihangnica*

## Abstract

**Background:**

Energy allocation between reproduction and survival represents a fundamental life-history challenge for animals in seasonal environments. Using integrated transcriptomics and metabolomics, we investigated *Nanorana taihangnica* (Anura: Dicroglossidae), a non-amplexus stream frog endemic to China, to elucidate the seasonal morphological and molecular coordination between the testis and abdominal fat body.

**Results:**

Morphological analysis showed that fat body adipocyte cross-sectional area minimized at the end of the breeding season but rapidly recovered thereafter, while testicular volume continued declining post-breeding and only recovered during the non-breeding period. During breeding season, multi-omics analyses revealed that the fat body enhanced fatty acid oxidation, upregulated histidine-carnosine metabolism, activated NAD^+^ metabolism and FOXO3-mediated antioxidative responses to mitigate metabolic stress, and regulated adipocyte survival and apoptosis via sphingolipid signaling. Seasonal testicular development was centrally regulated by the mTOR signaling pathway, whose activity integrated autophagy levels, NAD^+^ availability, and aspartate metabolism to coordinate spermatogonial proliferation and spermatogenesis.

**Conclusions:**

This study demonstrates that *N. taihangnica* optimizes seasonal energy storage, allocation, and reproductive investment through molecular and metabolic crosstalk between the fat body and testis, providing empirical insights into the physiological integration of life-history strategies in animals inhabiting fluctuating environments.

**Supplementary Information:**

The online version contains supplementary material available at https://doi.org/10.1186/s12864-026-13080-4.

## Background

The evolution of life history is driven by trade-offs in energy allocation among survival, growth, and reproduction [1]. In seasonal environments, periodic resource fluctuations intensify these trade-offs and raise a central challenge of how to tune the allocation of limited energy to environmental rhythms in order to maximize long-term fitness. This fundamental constraint shapes the evolution of complex life-history strategies, with seasonal reproduction representing a prominent adaptive response [2, 3]. In many species with complex social systems, males bear substantial reproductive costs, and their energy condition directly modulates reproductive success [4, 5].

The amphibian fat body (FB) functions as the primary organ for lipid storage, containing high levels of triglycerides and cholesterol. Its mobilization is regulated by the hypothalamus-hypophyseal system [6, 7]. Nutrients are delivered from the FB to the testes via direct vascular connections, supporting normal testicular physiology [8]. Experimental evidence demonstrates that FB removal causes rapid testicular atrophy, whereas administration of FB homogenate can restore testicular volume [9]. Furthermore, the FB provides energy for vocalization and courtship during the breeding season; severe depletion of its reserves leads to the cessation of reproductive activity [10]. Together, these findings establish the FB as a critical integrator of male reproductive function and energetic condition.

Amphibians represent a classic model of seasonal reproduction. In these species, the annual cycle of the male gonad (i.e., the testis) forms a crucial internal rhythm that synchronizes reproductive activity with optimal environmental conditions, thereby ensuring reproductive success and offspring survival [11]. Seasonal fluctuations in testicular weight and volume are driven by changes in spermatogenic activity [11–13]. This cyclical pattern is regulated by key environmental factors, including temperature, photoperiod, and humidity [12, 14–16]. These external signals are integrated and transduced through the hypothalamic-pituitary–gonadal (HPG) axis, which ultimately governs testicular function and maintains its annual periodicity [16].

*Nanorana taihangnica* (Anura: Dicroglossidae) is an endemic stream-dwelling frog inhabiting temperate zones with marked seasonal variation [17, 18]. Notably, through long-term observations, we have observed that males exhibit relatively large testes with considerable monthly variation in testicular volume. However, the spermatogenic pattern, the cyclical dynamics of testicular development, and how these processes coordinate with seasonal energy metabolism in the FB remain poorly understood. In this study, we systematically characterize the seasonal morphological changes in the testis and fat bodies of *N. taihangnica* and employ integrated transcriptomic and metabolomic analyses to identify the key molecular pathways underlying their synchronous seasonal variations. This work will elucidate the unique reproductive adaptations of *N. taihangnica* and provide new insights into how animals optimize energy allocation through integrated physiological and molecular responses to periodic environmental constraints.

## Results

### Seasonal variations in FB adipocyte size and testicular volume

**Fig. 1:**
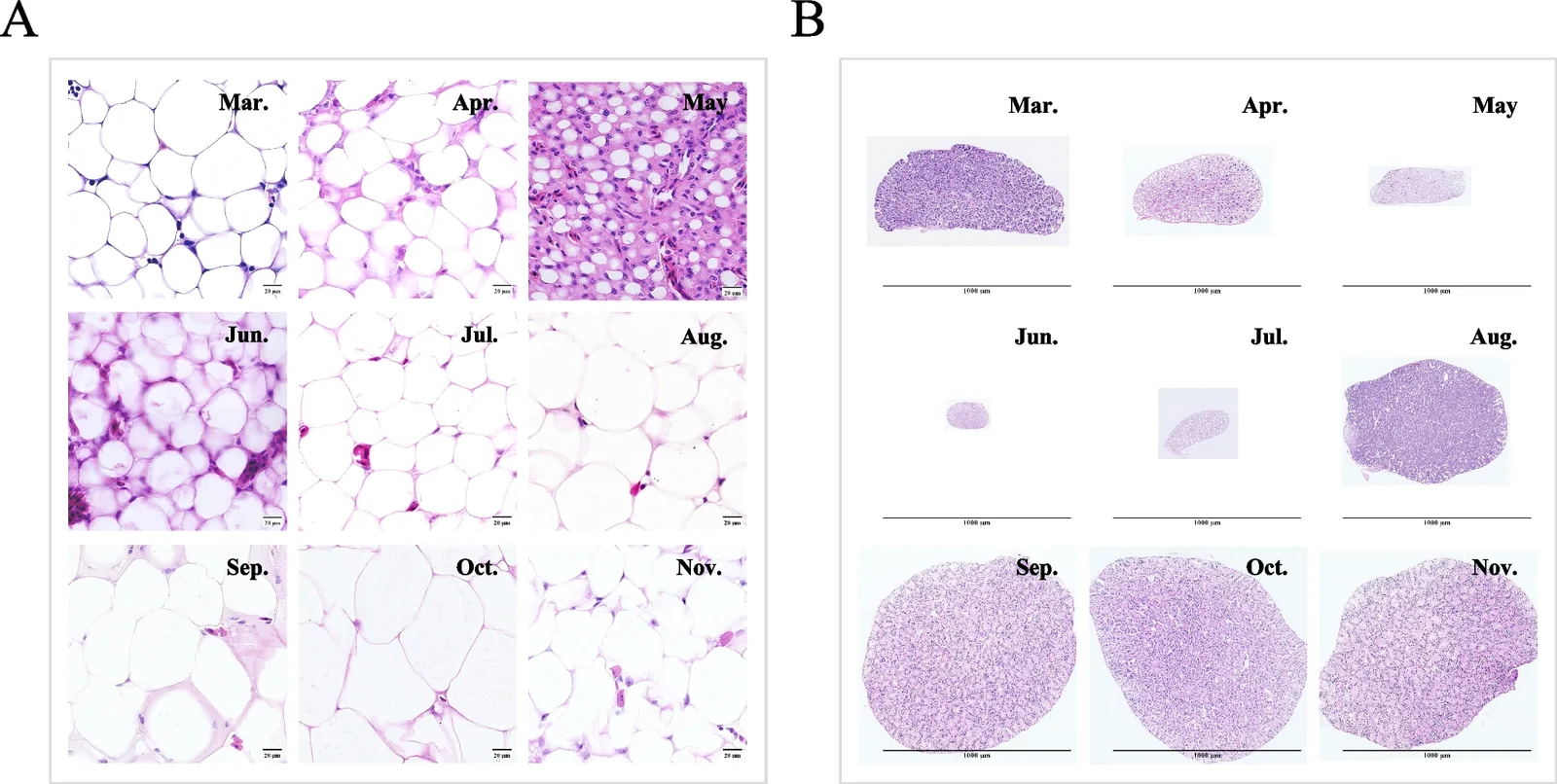
Seasonal changes of testis and FB in different months. **A** Histomorphology of FB in different months. **B** Histomorphology of testis in different months

The cross-sectional area of adipocytes in FB reached its lowest point between March and May, and increased progressively to peak in October, followed by a significant decrease in November (Fig. 1A). In contrast, testicular volume declined from March to June and subsequently increased, attaining its maximum in November (Fig. 1B).

### Seasonal changes of testicular histology and spermatogenesis

**Fig. 2:**
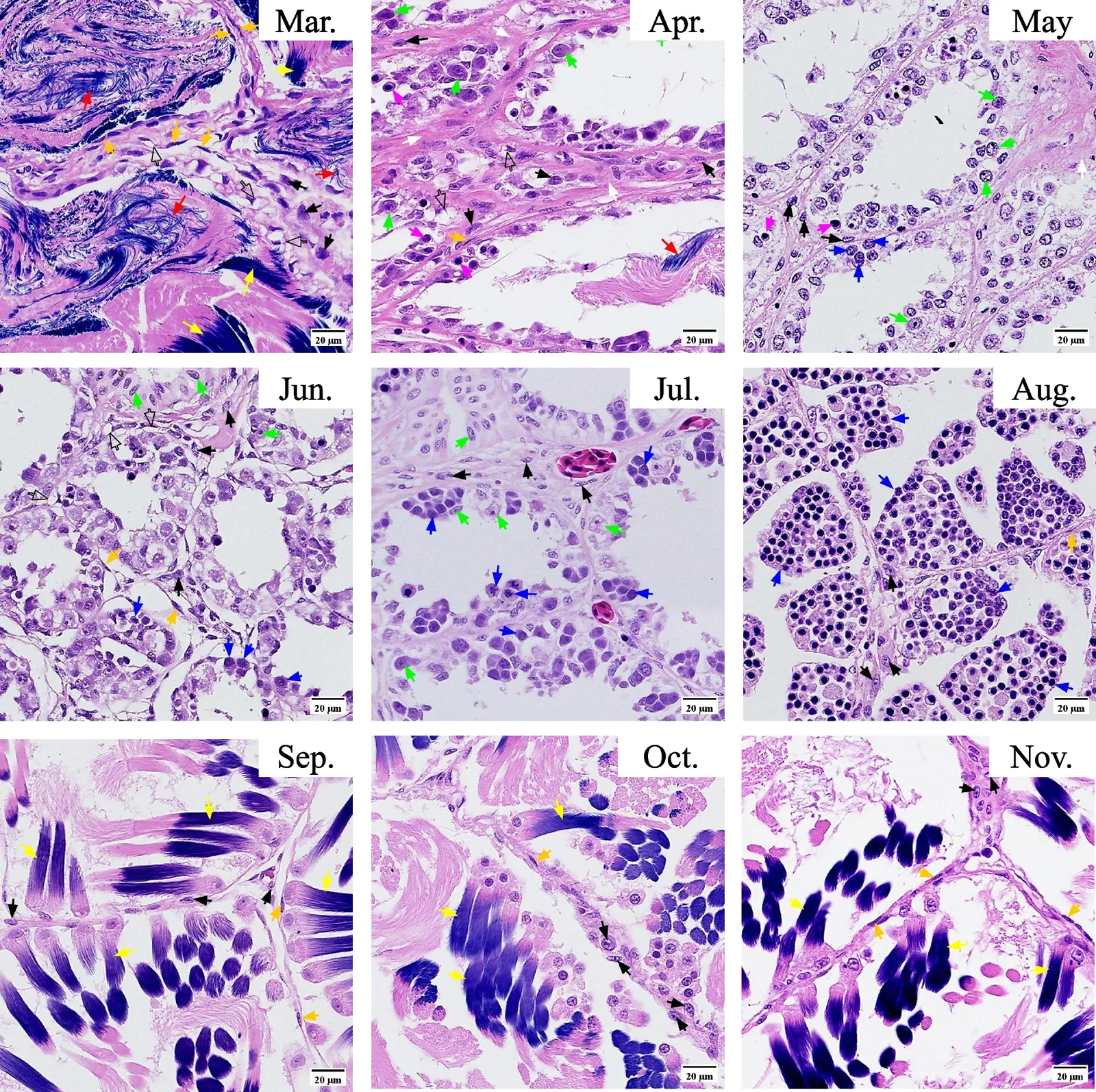
Seasonal differences in testicular cell types in male *Nanorana taihangnica*. Magenta arrow: spermatogonia, blue arrow: spermatocytes, yellow arrow: spermatids, red arrow: spermatozoa, orange arrow with black outline: myoid cell, green arrow: Sertoli cell, black arrow: Leydig cell, hollow arrow: adipocytes, white arrow, interstitial tissue

The spermatogenic cycle of male *N. taihangnica* was discontinuous, as revealed by histological examination of seminiferous tubule composition across different months (Fig. 2). In March (pre-breeding period), spermatids transitioned from bundled arrangements at the tubule base to a scattered distribution toward the center. During the breeding season, sperm content within the tubules varied considerably, even within the same individual, suggesting multiple sperm release events until depletion. From May to September (the post-breeding period), spermatids gradually formed within the seminiferous tubules and eventually became realigned at the base, displaying a sperm tract structure. Between September and November, the tubules entered a temporary dormant phase, during which the sperm bundle structure was maintained and the cross-sectional area remained unchanged. From June to August, no spermatogonia were observed; only spermatocytes were present, and their numbers increased monthly. Although amphibian spermatogenesis typically occurs within cyst structures, such structures were only observed in July and August in this study. Myoid cells were abundant and closely surrounded the seminiferous tubules in March. By April, atrophic tubules were enveloped by rapidly proliferating interstitial tissue, with fewer myoid cells compared to March. Myoid cells were still present in June and August but were absent in May or July. Leydig cells were observed throughout all months. Interstitial tissue content was significantly higher from April to July compared to other months, appearing as dense connective tissue. Additionally, cells with typical unilocular adipocyte morphology were observed in March, April and June. In March, adipocytes were densely distributed in the testicular stroma and near the tubule base. In April and June, they were located within leydig cell rich interstitial tissue.

### Transcriptome sequencing and DEGs identification

To exclude individual variation, samples from multiple specimens of the same season and tissue type were pooled. Specifically, two pooled samples from the April fat body (named TH4F1 and TH4F2), two from the August fat body (named TH8F1 and TH8F2), two from the April testis (named TH4T1 and TH4T2), and two from the August testis (named TH8T1 and TH8T2) of *N. taihangnica* were prepared for de novo transcriptome sequencing The quality of the raw sequencing data for each sample is summarized in Supplementary Table 1.

**Fig. 3:**
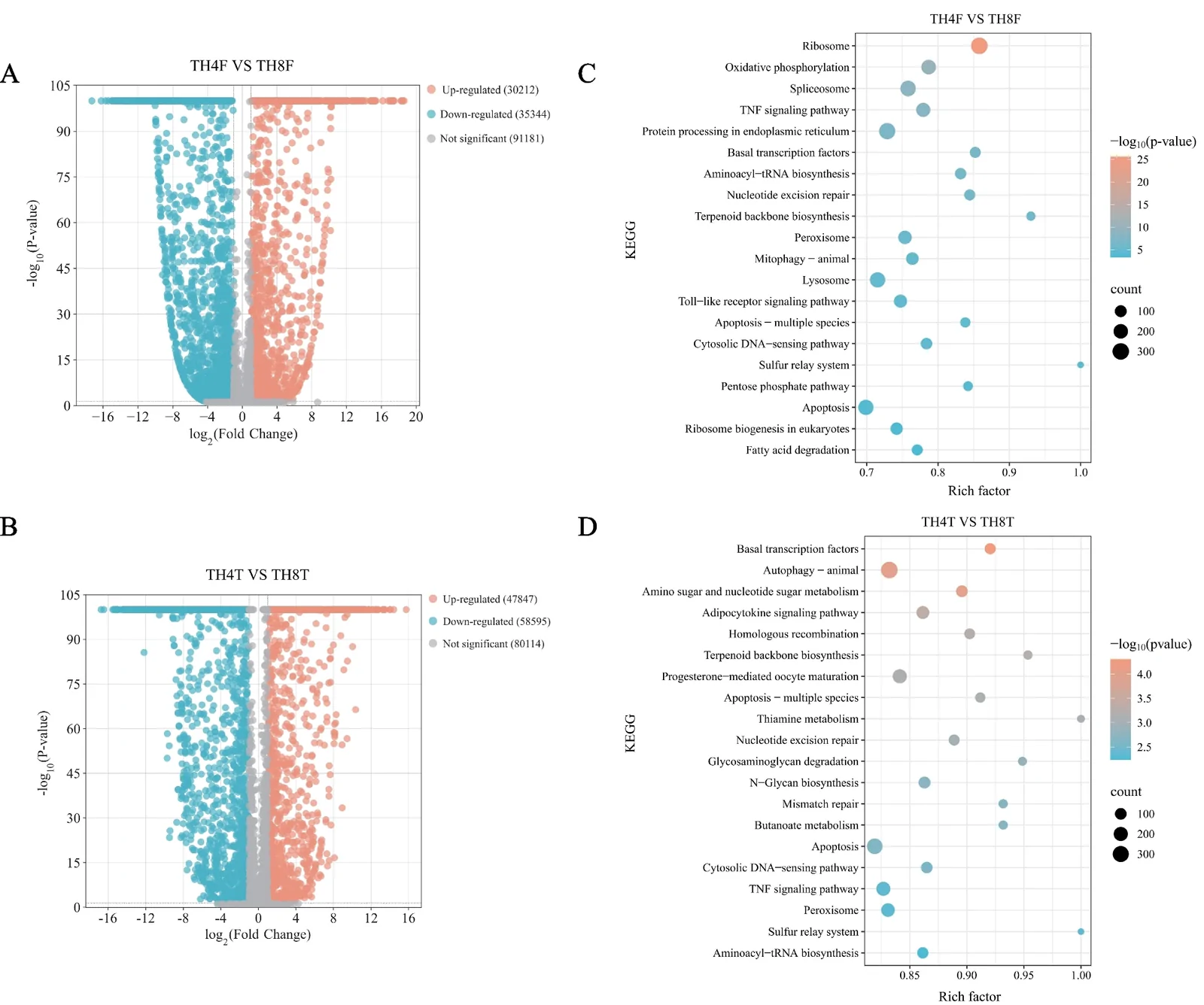
Statistical plots of differentially expressed genes and KEGG enrichment analysis. **A**, **B** DEG analysis across different seasons: Volcano plots for TH4F versus TH8F (**A**) and TH4T versus TH8T (**B**) (red: upregulated genes; blue: downregulated genes; gray: non-significant genes; thresholds: |log₂FoldChange|> 1, *p <* 0.05). The TOP 20 KEGG enrichment circles of differentially expressed genes in TH4F versus TH8F (**C**) and TH4T versus TH8T (**D**) are plotted. The X-axis of the bubble chart was rich factor. The greater the bubble size, the more the number of differential DEGs involved. Blue circle means high P values and red circle means low *P* values

After filtering, a total of 367,763,472 clean reads were obtained from the eight samples, corresponding to 55.35 Gb of clean data (Supplementary Table 2). The guanine-cytosine (GC) content across samples ranged from 44.60% to 46.40% (Supplementary Table 2). The clean reads were de novo assembled using Trinity to generate transcripts. The longest isoform per gene was selected as a representative unigene. Assembly statistics for both transcripts and unigenes are provided in Supplementary Table 3. Functional annotation of unigenes was performed against the NR, GO, KEGG, eggNOG, Swiss-Prot, and Pfam databases (Supplementary Table 4). Differential gene expression analysis, applying a threshold of |log2FoldChange|≥ 1 with a P-value < 0.05, identified 65,556 DEGs (30,212 up-regulated and 35,344 down-regulated) in the TH4F vs TH8F comparison, and 106,442 DEGs (47,847 up-regulated and 58,595 down-regulated) in the TH4T vs TH8T comparison. These DEGs are visualized in volcano and bar plots for the TH4F vs TH8F (Fig. 3A) and TH4T vs TH8T (Fig. 3B) comparisons, with detailed gene lists provided information in Supplementary Tables 5 and 6.

### KEGG pathways analysis in DEGs

The DEGs identified in this study were mapped to KEGG pathways for functional annotation. In the TH4F vs TH8F and TH4T vs TH8T comparisons, 65 and 44 KEGG pathways were significantly enriched (*p <* 0.05), respectively. The top 20 enriched KEGG pathways for each comparison are presented in enrichment circle plots (Fig. 3C and D), with full results provided in Supplementary Tables 7 and 8.

### Seasonal metabolite changes in FB

To account for individual variation, tissues from multiple *N. taihangnica* individuals were randomly pooled. Three biological replicates each of April fat body (named TH4F1-3), August fat body (named TH8F1-3), April testis (named TH4T1-3), and August testis (named TH8T1-3) were prepared for untargeted metabolomics analysis using multi-response multiple reaction monitoring (MRM) based on a locally constructed database. In the TH4F vs TH8F comparison, 929 metabolites spanning 20 categories were identified with major groups including lipids and lipid-like molecules (320, 34.4%), organoheterocyclic compounds (187, 20.1%), organic acids and derivatives (125, 13.5%), 90 benzenoids (90, 9.7%), 43 organic oxygen compounds (43, 4.6%), fatty acids (42, 4.5%), organic nitrogen compounds (29, 3.1%), amino acids and peptides (18, 1.9%), alkaloids (12, 1.3%), phenylpropanoids and polyketides (12, 1.3%), terpenoids (11, 1.2%), carbohydrates (10, 1.2%), and other metabolites (3.2%) (Supplementary Fig. 1 A). For the TH4T vs TH8T comparison, 1340 metabolites from 20 categories were identified. The predominant categories comprised lipids and lipid-like molecules (335, 25.0%), 287 organoheterocyclic compounds (287, 21.4%), 252 organic acids and derivatives (252, 18.8%), benzenoids (149, 11.1%), 54 organic oxygen compounds (54, 4.0%), fatty acids (42, 3.1%), 34 organic nitrogen compounds (34, 2.5%), phenylpropanoids and polyketides (25, 1.9%), carbohydrates (25, 1.9%), nucleosides, nucleotides, and analogues (21, 1.6%), alkaloids (19, 1.4%), shikimates and phenylpropanoids (13, 1.0%), and other metabolites (2.5%) (Supplementary Fig. 1B). The metabolite profiles of the TH4F vs. TH8F and TH4T vs. TH8T groups were further examined using principal component analysis (PCA) and orthogonal partial least squares-discriminant analysis (OPLS-DA). The score plotS (Supplementary Figs. 2 and 3) revealed clear separation between these groups, indicating significant seasonal differences in the metabolite composition of *N. taihangnica*.

### Analysis of differential metabolites and KEGG pathway enrichment

**Fig. 4:**
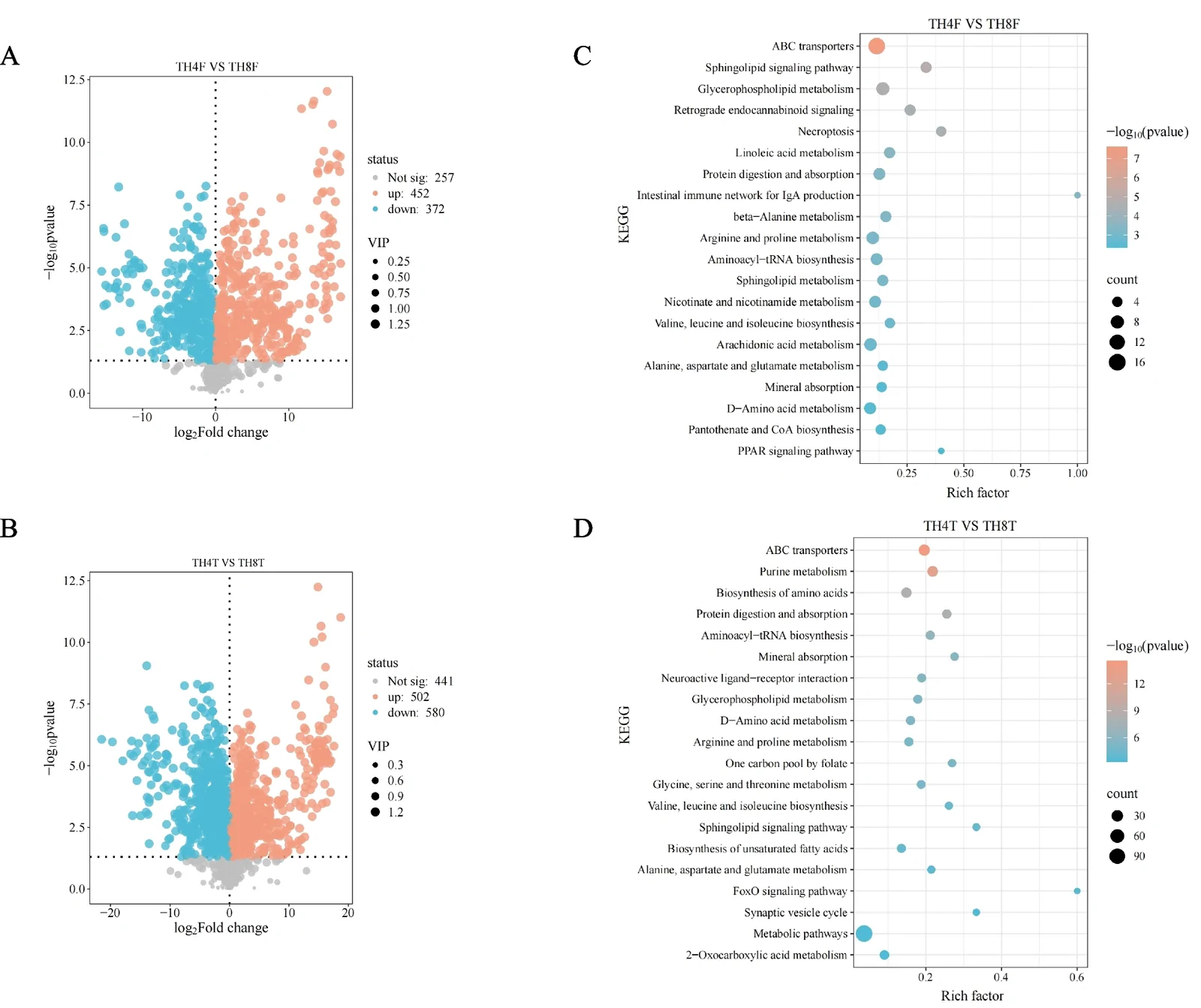
Changes in DEMs expression and pathway enrichment in different seasons. Volcano plots for TH4F versus TH8F (**A**) and TH4T versus TH8T (**B**) (red: upregulated metabolites; blue: downregulated metabolites; gray: non-significant metabolites. Bubble plots of the TOP 20 KEGG enrichment results for DEMs in TH4F versus TH8F (**C**) and TH4T versus TH8T (**D**). The X-axis of the bubble chart was rich factor. The greater the bubble size, the more the number of differential DEMs involved. Blue circle means high P values and red circle means low *P* values

To characterize seasonal effects on the metabolites, differential metabolites (DEMs) were identified using the criteria of variable importance in projection (VIP) ≥ 1 and *P* value < 0.05. The TH4F vs. TH8F comparison revealed 452 upregulated metabolites and 372 downregulated metabolites (Fig. 4A, Supplementary Table 9), while the TH4T vs. TH8T comparison identified 502 upregulated and 580 downregulated metabolites (Fig. 4B, Supplementary Table 10). KEGG pathway enrichment analysis Showed that DEMs mapped to 123 pathways in the FB comparison, with 43 significantly enriched (*p <* 0.05); the top 20 pathways are shown in Fig. 4C and Supplementary Table 11. In the testis comparison, DEMs mapped to 138 KEGG pathways, with 61 showing significant enrichment (*p <* 0.05); the top 20 pathways are presented in Fig. 4D and Supplementary Table 12.

### Transcriptomic and metabolomic co-analysis

**Fig. 5:**
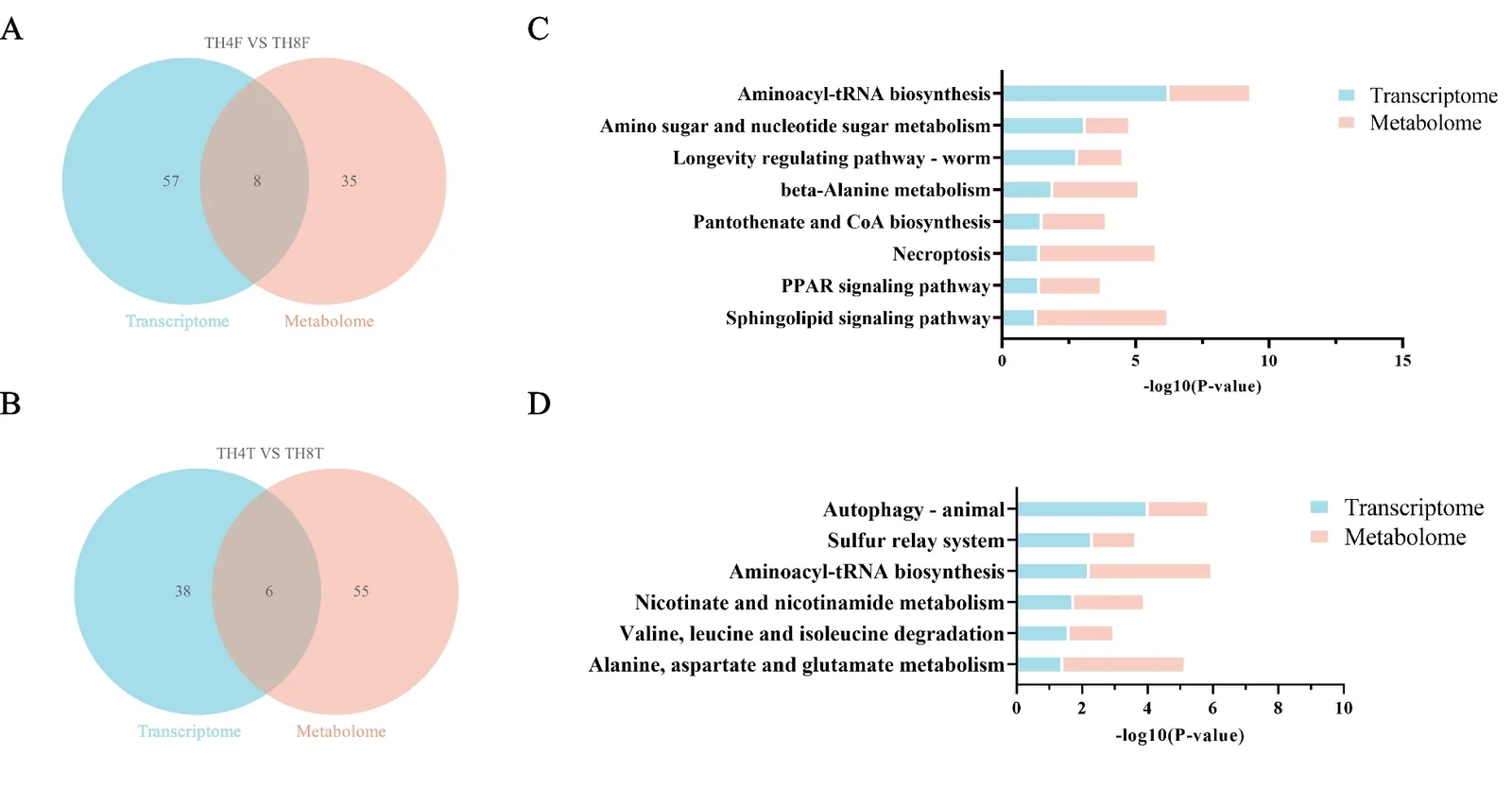
Transcriptomic and metabolomic co-analysis. Venn diagrams illustrate the number of significantly enriched KEGG pathways identified from transcriptomic and metabolomic analyses in the TH4F versus TH8F (**A**) and TH4T versus TH8T (**B**) comparisons. Pathways significantly enriched in the transcriptomic KEGG analysis are represented in blue, while those from the metabolomic KEGG analysis are shown in red. Bar graphs display the p-values of KEGG pathways derived from transcriptomic and metabolomic co-analysis for the TH4F versus TH8F (**C**) and TH4T versus TH8T (**D**) comparisons

**Fig. 6:**
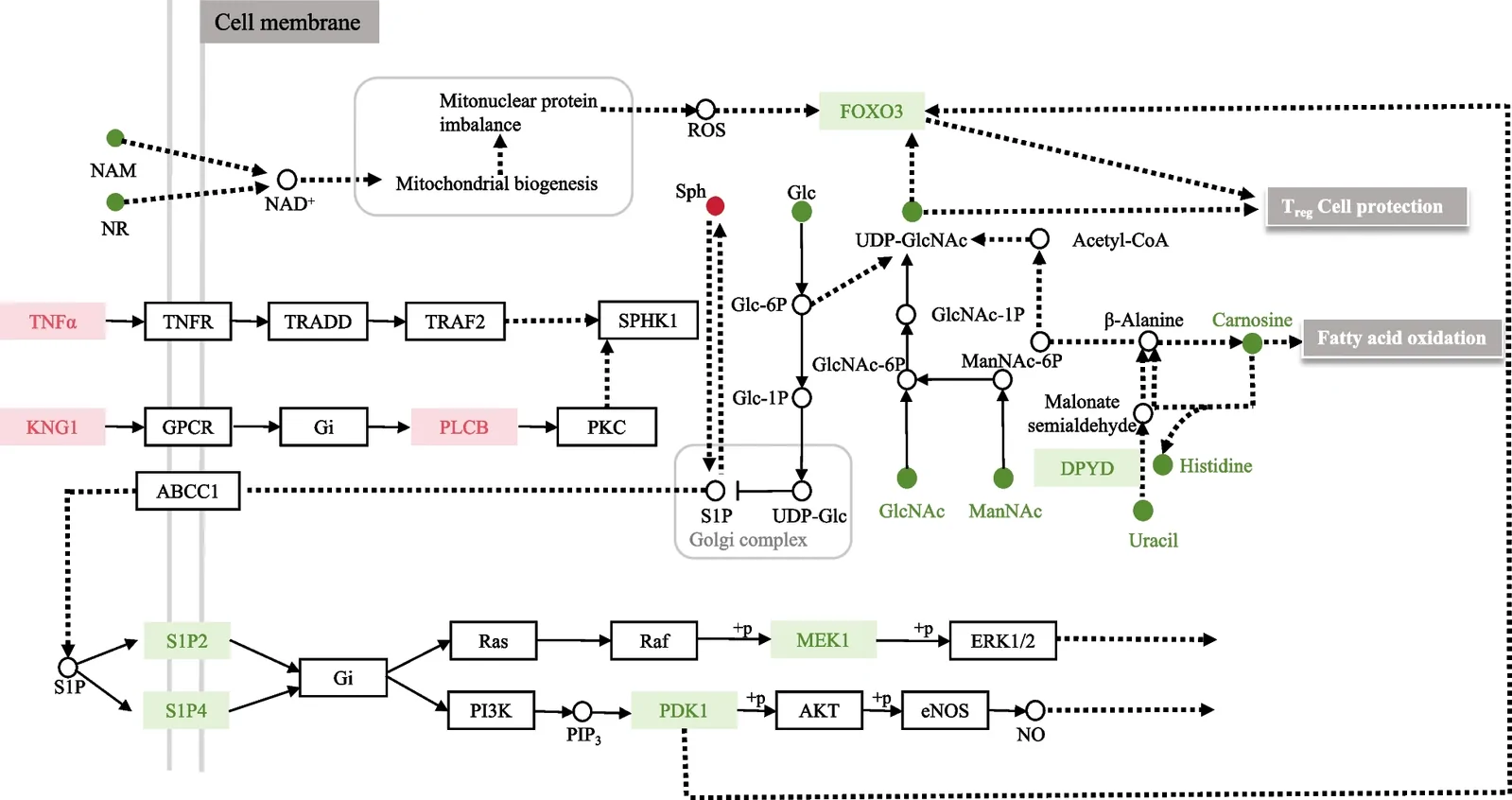
Schematic diagram of the integrated transcriptomic and metabolomic regulatory mechanism of commonly enriched pathways in adipose tissue between breeding and non-breeding periods. Boxes represent differentially expressed genes (DEGs): Red text indicates upregulated DEGs, and green text indicates downregulated DEGs in TH4F versus TH8F. Circles represent differentially expressed metabolites (DEMs): Red circles indicate upregulated DEMs, and green circles indicate downregulated DEMs in TH4F compared with TH8F

**Fig. 7:**
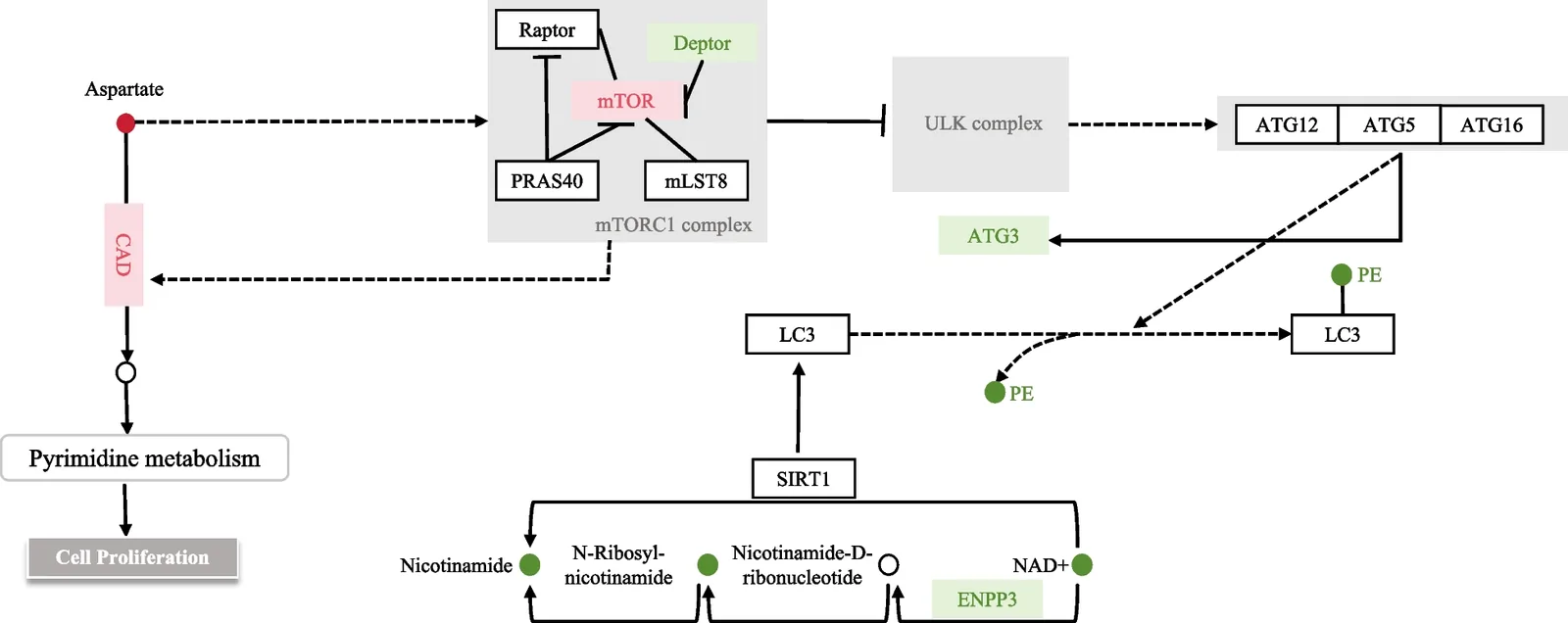
Schematic diagram of the integrated transcriptomic and metabolomic regulatory mechanism of commonly enriched pathways in testis between breeding and non-breeding periods. Boxes represent differentially expressed genes (DEGs): Red text indicates upregulated DEGs, and green text indicates downregulated DEGs in TH4T versus TH8T. Circles represent differentially expressed metabolites (DEMs): Red circles indicate upregulated DEMs, and green circles indicate downregulated DEMs in TH4T compared with TH8T

To further elucidate the seasonally regulated biological pathways, KEGG enrichment results from transcriptomic and metabolomic analyses were compared. Shared pathways were visualized in Venn diagrams (Fig. 5A, B) and presented in bar charts, with eight common pathways in the TH4F vs. TH8F comparison (Fig. 5C) and six in the TH4T vs. TH8T comparison (Fig. 5D). Among the commonly enriched pathways in the TH4F vs. TH8F comparison, the PPAR signaling pathway and the sphingolipid signaling pathway were linked to lipid metabolism. Key differentially expressed genes (DEGs) and DEMs within these commonly enriched pathways were integrated to generate a proposed seasonal regulatory mechanism for the FB (Fig. 6). Similarly, In the TH4T vs. TH8T comparison, the autophagy-animal pathway and nicotinate and nicotinamide metabolism pathway showed direct relevance to testicular function. The associated DEGs and DEMs were synthesized to construct a seasonal regulatory mechanism diagram for the testis (Fig. 7).

### Validation of DEGs by qRT-PCR

To validate the reliability of the transcriptome data, six DEGs were verified by qRT-PCR. These included three genes related to FB variation (Supplementary Fig. 4 A) and three related to testis variation (Supplementary Fig. 4B). The results showed a high degree of consistency between qRT-PCR and RNA sequencing (RNA-seq) data (Supplementary Fig. 4 C, 4D), confirming the accuracy of our RNA-seq data.

## Discussion

### High energy demands in breeding season and FB antioxidative protection under high fatty acid oxidation state

Morphological analysis revealed that the cross-sectional area of adipocytes in male *N. taihangnica* was minimal at the end of the breeding season (May) and increased rapidly to higher levels by June. In contrast, testicular volume continued to decline after breeding (June) and did not begin to recover until July. These findings suggest that the FB plays an essential role in supporting reproductive behavior and subsequent testicular development in males. Furthermore, lipid content in the male FB decreased significantly in November compared to October, reflecting its function in providing energy for physiological maintenance under food limited winter conditions. Together, these results demonstrate the dual functional adaptation of the FB in supporting reproduction and environmental stress responses.

Numerous studies have demonstrated that peripheral L-histidine regulated lipid metabolism through multiple mechanisms. A key mechanism is its conversion to histamine, which activates hypothalamic histaminergic neurons to suppress feeding, as confirmed in both human and animal models [19, 20]. Additionally, histidine promotes lipolysis in visceral white adipose tissue via sympathetic nervous system activation; for instance, intraperitoneal histidine injection significantly enhances sympathetic nerve activity in epididymal adipose tissue while increasing glycerol and free fatty acid levels [21]. The physiological relevance is further supported by histidine decarboxylase (HDC) knockout animals, which exhibit marked visceral fat accumulation and weight gain [22]. Human epidemiological data further support this role: dietary histidine intake correlates inversely with BMI, waist circumference, and blood pressure in overweight/obese adults [23], and plasma histidine levels are significantly lower in obese and type 2 diabetic adolescents [24]. Collectively, these findings indicate that histidine (precursor of histamine) plays an essential role in both central appetite regulation and peripheral lipolysis. Carnosine, a histidine-containing dipeptide, also critically modulates metabolic regulation. Synthesized from β-alanine and L-histidine, carnosine synthesis can be constrained by histidine availability despite β-alanine being the rate-limiting precursor [25]. Carnosine administration increases sympathetic nerve activity and promotes visceral white adipose tissue lipolysis in rats [26, 27]. Furthermore, carnosine reduces hepatic oxidative stress and triglyceride levels in diet-induced metabolic dysfunction-associated steatotic liver disease (MASLD) rat model, accompanied by significant upregulation [28]. Our metabolomic analysis revealed significantly elevated histidine and carnosine levels during the breeding season, along with increased uracil (a β-alanine precursor) and dihydropyrimidine dehydrogenase (DPYD) activity [29], supporting enhanced synthesis of both histidine and carnosine during this period. These results indicate that the adipose tissue of male *N. taihangnica* undergoes elevated energy expenditure and fatty acid oxidation during the breeding season compared to the non-breeding period, which aligns with the observed minimum adipocyte cross-sectional area during this reproductive phase.

Studies have shown that upregulation of fatty acid oxidation induced by PPARγ agonist promotes the entry of fructose-6-phosphate into the hexosamine biosynthesis pathway (HBP), thereby elevating UDP-GlcNAc levels. Increased UDP-GlcNAc enhances transcription of the *FOXO3* (Forkhead box O3) gene, a key regulator of regulatory T cells (Treg) development and function [30–32]. In adipose tissue, Tregs are essential for maintaining metabolic and immune homeostasis. They secrete anti-inflammatory factors and express immunosuppressive molecules to suppress the infiltration and activity of pro-inflammatory immune cells, reducing the release of pro-inflammatory cytokines and thereby alleviating local adipose tissue inflammation. Fatty acid oxidation serves as the primary energy source for Tregs, and their metabolic state directly influences their anti-inflammatory function [32–34]. In this study, transcriptomic analysis revealed significant *FOXO3* gene differential expression, while metabolomics showed marked alterations in UDP-GlcNAc and its precursors (GlcNAc and ManNAc). These findings suggest that high fatty-acid oxidation in the FB of *N. taihangnica* during the reproductive period (driven by high energy demands) promotes Treg differentiation in adipose tissue. This mechanism likely helps counteract inflammation induced by elevated fatty-acid oxidation, thereby ensuring the maintenance of immune homeostasis.

During the breeding period, elevated fatty-acid oxidation imposes significant metabolic stress on adipose tissue. Nicotinamide (NAM), a key intermediate in NAD^+^ metabolism, serves both as the primary byproduct of NAD^+^-consuming enzymatic reactions and as a core precursor for the NAD^+^ salvage synthesis pathway. Nicotinamide riboside (NR), another critical NAD^+^ precursor, is phosphorylated by nicotinamide riboside kinase (NRK) to generate nicotinamide mononucleotide (NMN), ultimately contributing to NAD^+^ regeneration as an essential substrate in the salvage pathway [35, 36]. Under metabolic stress, NAD^+^ is catabolized by NAD^+^-consuming enzymes, releasing NAM, while NR enhances NAD^+^ levels via increased exogenous uptake or endogenous conversion, thereby counteracting oxidative stress induced by excessive energy expenditure and reducing inflammatory factors such as TNFα [36–38]. FOXO3, an evolutionarily conserved member of the forkhead box transcription factor family, regulates gene expression by binding specific DNA sequences and mediates cellular responses to oxidative stress [39, 40]. Studies demonstrate that elevated NAD^+^ levels upregulate *FOXO3* expression, significantly enhancing antioxidant defenses [41]. As a metabolic signaling molecule, NAD^+^ is activated under metabolic stress to mitigate oxidative damage [35–37]. In this study, metabolomic analysis revealed high expression of NAM and NR in the adipose tissue of male *N. taihangnica* during the breeding season, while transcriptomic data indicated upregulation of the *FOXO3* gene. These findings suggest a coordinated adaptive mechanism under high fatty acid oxidation-induced metabolic stress. Specifically, NAD^+^ depletion by NAD^+^-consuming enzymes generates NAM, and its accumulation promotes oxidative stress. In contrast, NR-mediated NAD^+^ resynthesis, via either endogenous production or exogenous uptake, activates *FOXO3* expression to counteract oxidative stress.

Adipose tissue adapts to seasonal changes in energy demand through diverse regulatory mechanisms, with the sphingolipid signaling pathway acting as a key sensor in this adaptive process. TNFα, a pleiotropic cytokine involved in systemic inflammation and immune regulation, plays a critical role in modulating sphingolipid signaling [42, 43]. Studies indicate that, upon TNFα and other agonists stimulation, SPHK1 phosphorylates sphingosine (Sph) to generate sphingosine-1-phosphate (S1P) through a TNFα receptor-dependent activation mechanism [43–45]. S1P suppresses apoptosis by activating anti-apoptotic mechanisms [46] and inhibiting ceramide-mediated programmed cell death [47]. In this study, elevated *TNFα* gene expression in the adipose tissue of *N. taihangnica* during August was accompanied by increased Sph and reduced ceramide levels, suggesting enhanced anti-apoptotic activity. This aligns with the established activation mechanism of the sphingolipid pathway, in which PKCε and GPCR ligands promote SPHK1-mediated S1P production [48]. Transcriptomic data further revealed an upregulation of *KNG1*, a GPCR ligand, in August and *KNG1* may indirectly activate PKCε via *PLCB* [49],—+ thereby facilitating Sph-to-S1P conversion and supporting the hypothesis of elevated S1P levels during this period. Although S1P promotes adipocyte survival through anti-apoptotic mechanisms, controlled apoptosis remains essential for adipose tissue homeostasis. First, adipocyte apoptosis helps limit fat storage capacity, thereby preventing excessive tissue expansion [50]. Second, it contributes to postnatal adipose tissue remodeling, supporting structural maturation and cellular turnover [51]. Additionally, UDP-glucose (UDPG), a key intermediate in glycogen synthesis, has been shown to inhibit lipogenesis by suppressing S1P in the liver [52]. The low UDPG levels observed in August (the non-breeding season) suggest active lipid synthesis, which was consistent with morphological findings in this study.

The upregulation of *S1P2* and *S1P4* during the breeding season indicates that extracellular S1P predominantly regulates adipose tissue in this period. S1PR2, a Gi-coupled receptor, activates PI3K and MAPK cascades to promote cell survival [53, 54]. Although S1P is synthesized intracellularly, its extracellular levels are low; intracellular S1P is exported to exert its effects via S1P receptors in various physiological processes [55, 56]. PDK1, a key kinase in the PI3K/Akt pathway, drives cell proliferation and survival [57], while MEK1, a essential component of the MAPK pathway, regulates proliferation, survival, and mitosis and its loss leads to G2 arrest and apoptosis [58]. The PI3K/Akt and MAPK pathways cooperate to modulate cell proliferation and differentiation. PDK1 enhances MAPK/ERK signaling via PKC and directly phosphorylates MEK1, which then activates ERK to promote proliferation and differentiation [59]. While S1P2-mediated PI3K/Akt activation is well-established, the role of S1P4 in PI3K/Akt or MAPK signaling remains unclear. Here, the concurrent elevation of *S1P2* and *S1P4* expression during the breeding season suggests that S1P4 may complement S1P2-dependent activation, with the PI3K/Akt and MAPK pathways synergistically driving adipocyte proliferation and differentiation. This mechanism is further supported by morphological data, which show that adipocyte cross-sectional area recovers rapidly after reaching a seasonal minimum, reflecting the molecular adaptability of adipose tissue to cyclical changes. In agreement with these pathway-level findings, qRT-PCR validation confirmed the upregulation of *FOXO3* and *PDPK1* in the FB during the breeding season, supporting the activation of antioxidative and PI3K/Akt-mediated survival signaling, respectively. The concurrent upregulation of PLCB3 further corroborates the involvement of GPCR-PLC-PKC signaling in the seasonal regulation of the sphingolipid pathway.

### mTOR acts as a key regulator of the testicular cycle in *N. taihangnica*

The mechanistic target of rapamycin (mTOR), an evolutionarily conserved serine/threonine protein kinase, belongs to the phosphoinositide 3-kinase-related kinase (PI3KK) superfamily and serves as a central regulator of cellular metabolism and growth. mTOR functions as the catalytic core within two distinct complexes, i.e., mTOR complex 1 (mTORC1) and mTOR complex 2 (mTORC2). mTORC1 is a key regulator of autophagy and negatively modulates LC3-mediated autophagic processes by inhibiting ULK1 complex activity and suppressing LC3 processing and localization. It also integrates intracellular and extracellular energy and nutrient signals to coordinate the initiation and progression of autophagy [60–63]. In the testis, mTOR signaling is essential for spermatogenesis. In spermatogonia, mTOR supports differentiation, and its inhibition impairs differentiation, suppresses proliferation, and reduces sperm production [64, 65]. In Sertoli cells, mTOR helps maintain seminiferous epithelium structure and cell polarity while reducing germ cell apoptosis. Conditional knockout of *Mtor* in Sertoli cells leads to disorganized seminiferous epithelium, loss of polarity, degeneration of pachytene spermatocytes and spermatids, increased germ cell apoptosis, and ultimately spermatogenic failure [66]. Furthermore, mTOR and its downstream signaling contribute to cellular homeostasis during spermiogenesis. For instance, in the Chinese soft-shelled turtle, elevated autophagy, indicated by increased LC3 expression, promotes spermatid maturation and acrosome formation, whereas autophagy suppression leads to sperm malformations [67]. In aging roosters, inhibiting testicular mTOR activity and enhancing LC3 deacetylation improves sperm motility [68]. Notably, mTOR expression and function exhibit spatiotemporal and species-specific variation. In *Bison bonasus*, testicular mTOR mRNA levels are significantly higher before and after the breeding season than during the breeding season [69]. In *Lagostomus maximus*, LC3 is nearly absent in the testis during the non-breeding season, increases gradually during the transitional to the breeding season, and becomes highly expressed during active spermatogenesis [70]. In SD rats, mTOR expression in spermatogonia declines progressively with age [65].

In our study, the expression of mTOR and its downstream effector ATG3, together with the metabolite phosphatidylethanolamine (PE), showed marked seasonal variation, suggesting a potential role for mTOR in orchestrating the cyclical spermatogenesis of *N. taihangnica*. The low autophagic activity observed during the post-breeding phase is temporally associated with the period of spermatogonial differentiation and spermatocyte development, whereas the relatively higher autophagy levels during the breeding season coincide with a supportive role in spermiogenesis, the maintenance of sperm viability, and the elevated energy demands of testicular activity during this period. Beyond energy and nutrient signaling, autophagy is regulated by additional pathways. Notably, the NAD^+^-dependent deacetylase SIRT1 (Silent Information Regulator 1) [71–73] modulates autophagy and other biological processes by utilizing NAD^+^ as an essential cofactor [74]. It has been demonstrated that SIRT1 in mouse Sertoli cells promotes autophagosome formation and supports testosterone synthesis in Leydig cells via deacetylation of LC3 [75]. Our results revealed that both NAD^+^ and nicotinamide were significantly downregulated in August, corresponding to the non-breeding season. This reduction in NAD^+^ availability likely impairs SIRT1-mediated deacetylation of LC3, leading to decreased autophagic activity, which was consistent with the concurrently observed reduction in PE levels. Furthermore, qRT-PCR validation confirmed the differential expression of ENPP3, a gene involved in NAD^+^ metabolite interconversion, providing additional evidence that NAD^+^ metabolism participates in the seasonal regulation of testicular autophagy. Collectively, these seasonal molecular changes are suggestive of a species-specific reproductive strategy shaped by precise regulation of energy allocation across different phases of the spermatogenic cycle. Consistent with the transcriptomic findings, qRT-PCR validation confirmed the upregulation of *mTOR* and the concomitant downregulation of its endogenous inhibitor *DEPTOR* in the testis during the breeding season. This reciprocal expression pattern reinforces the interpretation that mTOR signaling is activated to support spermatogenesis and autophagy during this period.

Meanwhile, Oberkersch et al. [76] recently showed that in endothelial cells aspartate is a direct activator of mTORC1, which then phosphorylates CAD (Carbamoyl-phosphate synthetase 2, Aspartate transcarbamylase, and Dihydroorotase) to accelerate de novo pyrimidine synthesis and satisfy the DNA demands of rapid proliferation. In the present study, CAD activity rose in parallel with mTORC1 signaling in the testis of *N. taihangnica* as the breeding season commenced. We therefore propose that the aspartate that accumulates in the post-breeding gonad serves as an upstream signal that reignites mTORC1. Once reactivated, mTORC1 phosphorylates and thereby boosts CAD, driving de novo pyrimidine production. We suggest that this molecular cascade supplies the necessary DNA precursors to support the extensive proliferation of spermatogonia, ensuring the proper progression of spermatogenic differentiation.

To further validate the seasonal periodicity of spermatogenesis observed in our morphological analysis, we examined the expression profiles of key stage-specific marker genes. In vertebrates, the highly conserved *PLZF* gene is a core factor in maintaining spermatogonial stem cell (SSC) characteristics [77]. Animal studies have shown that *PLZF* maintains the undifferentiated state of SSCs and ensures their long-term self-renewal capacity, and its expression is markedly downregulated upon SSC entry into differentiation [78, 79]. In our study, PLZF expression in *N. taihangnica* fluctuated dynamically across spermatogenic stages. Specifically, expression during the breeding season (April) was significantly higher than in the non-breeding season (August), a pattern highly consistent with our morphological observations (Supplementary Fig. 5). Similarly, in mammals, ID4 is specifically expressed in SSCs and serves as a core "switch" that determines cell fate during adult spermatogenesis. Under the regulation of niche signals, ID4 drives long-term SSC self-renewal and prevents premature differentiation [80]. Upon initiation of spermatogonial differentiation, ID4 expression is markedly downregulated and gradually disappears [81, 82]. Furthermore, aberrant continuous expression of ID4 severely impairs the development of spermatogonial progenitors and blocks their transition to a differentiated state, ultimately resulting in complete spermatogenic arrest and male infertility [80]. Our findings showed that ID4 expression in *N. taihangnica* also fluctuated across spermatogenic stages. Similar to *PLZF*, *ID4* expression was significantly higher during the breeding season than in the non-breeding season, further corroborating our histological staging (Supplementary Fig. 5).

We acknowledge several limitations of the present study that should be addressed in future work. First, while our results revealed a temporal association between mTOR pathway activity and seasonal variation in spermatogenesis, the causal relationships (including the proposed role of aspartate as an upstream activator of Mtorc1) require further functional validation. Second, although the expression profiles of PLZF and ID4 strongly supported our qualitative staging, our description of the periodicity of spermatogenesis lacks precise quantitative histological staging. Future studies should explicitly quantify the proportions of spermatogonia, spermatocytes, spermatids, and spermatozoa at each seasonal time point and directly correlate these parameters with mTOR pathway activity to establish a more definitive link. Third, due to the complex cellular composition of the testis, our bulk tissue analyses cannot determine the cell type-specific localization (e.g., in spermatogonia, Sertoli cells, or Leydig cells) of the observed seasonal variations in mTOR activity, autophagy, NAD^+^ levels, and CAD activity. This lack of cellular resolution limits deeper mechanistic insight into the stage-specific roles of this pathway in distinct spermatogenic events, such as germ cell proliferation, meiosis, and spermiogenesis.

## Conclusion

This study employed an integrated transcriptomic and metabolomic approach to systematically reveal the coordinated seasonal variations occurring between the testes and abdominal FB in *N. taihangnica*. Our results indicated that during the breeding season, the FB shifts to a state characterized by enhanced fatty acid oxidation, increased lipolysis via the histidine-carnosine metabolic pathway, and concurrent activation of NAD^+^ metabolism supported by FOXO3-mediated antioxidative protection to meet elevated energy demands. Moreover, the sphingolipid signaling pathway helps maintain adipose homeostasis by balancing cell survival and apoptosis during rapid energy mobilization. In the testes, cyclical development is centrally regulated by the mTOR signaling pathway, whose activity is linked to autophagy levels, NAD^+^ availability, and aspartate metabolism, thereby coordinating spermatogonial proliferation, differentiation, and spermiogenesis across seasons. Together, these findings elucidate, at the molecular level, how *N. taihangnica* achieves a seasonal balance between reproductive investment and somatic maintenance through precise energy allocation and metabolic adaptation. This work will provide new insights into the integrated physiological and molecular mechanisms underlying animal responses to periodic environmental challenges.

## Materials and methods

### Ethics statement

All animal procedures were conducted in strict accordance with China’s national ethical guidelines for laboratory animals and all applicable animal welfare regulations. The experimental protocol was reviewed and approved by the Institutional Animal Care and Ethics Committee of Henan Normal University (Approval No. HNSD-2026–04–062).

### Animals and sample preparation

Male *N. taihangnica* specimens were obtained from the Biological Resources Museum of Henan Normal University. These individuals were originally collected from the Taihang Mountain Macaque National Nature Reserve of Henan Province, North China, between 2010 and 2017. Males were identified in the field by the dense warty granules around the cloacal region during the breeding season, or by gonadal examination after euthanasia during the non-breeding season. Immediately after capture, euthanasia was performed and adipose tissue and testicular tissue were immediately collected. Euthanasia was humanely performed via immersion in a 1% aqueous solution of tricaine methanesulfonate (MS-222, Sigma-Aldrich), ensuring loss of consciousness prior to tissue collection. FB and testis tissue processing followed standardized protocols: one lateral FB and one testis were fixed in 4% paraformaldehyde and Bouin’s solution, respectively, then paraffin embedding, sectioned at 4-μm, and stained with hematoxylin–eosin (HE) for histological examination. The contralateral tissues were flash-frozen in liquid nitrogen and stored at −80 °C for subsequent molecular analyses. Snout—vent length (SVL) was measured to the nearest 0.01 mm using a vernier caliper. Testicular volume was calculated with the ellipsoid formula V = 4/3 × π × a × b^2^, where a and b represent the semi-major and semi-minor axes, respectively [83]. Cross-sectional area of cells in histological sections was measured using ImageJ software. For morphological analysis (i.e., testicular volume, adipocyte cross-sectional area, and histological observations), specimens were collected between 2010 and 2017 to characterize the annual cyclical changes of the testis and FB in this species. Because specimen collection in this study spanned several years (2010–2017), the potential impacts of interannual climate variations need to be addressed. Notably, Green et al. (2017) demonstrated that detectable climate-driven phenological shifts in amphibians typically require multi-decadal to centennial observation windows [84]. Furthermore, short-term temperature fluctuations have been shown to exert limited or counterintuitive effects on amphibian reproductive timing [85–87]. To verify local conditions, we fitted generalized linear models (GLMs) to the interannual trends of March–November mean temperature, minimum temperature, maximum temperature, and mean precipitation during 2010–2017, using climate data from the CRU TS dataset. No statistically significant trend was found for any of these climatic variables (Supplementary Fig. 6). Additionally, our sampling timeline was aligned with the species' documented reproductive cycle [88], minimizing environmental interference on the physiological states of specimens during specific phenological phases. For RNA sequencing (RNA-seq) and untargeted metabolomics, a total of 12 adult individuals, all collected in the same year (six sampled in April and six in August) were randomly assigned to four experimental groups based on season and tissue type: April fat body (TH4F), August fat body (TH8F), April testis (TH4T), and August testis (TH8T). Samples collected in April correspond to the breeding season, while those from August correspond to the non-breeding season.

### Transcriptomic experimental methods

Total RNA was extracted from the collected *N. taihangnica* samples using the TRIzol reagent (Invitrogen, CA, USA) following the manufacturer’s instruction. RNA quality and concentration were assessed with a NanoDrop 2000 spectrophotometer (Thermo Fisher Scientific, MA, USA), and integrity was examined by 1% agarose gel electrophoresis. For library construction, ≥ 1 μg of total RNA per sample was used as input with the NEBNext Ultra II RNA Library Prep Kit for Illumina (NEB, USA). Polyadenylated mRNA was enriched using Oligo(dT) magnetic beads and then fragmented by incubation with divalent cations at elevated temperature. First-strand cDNA was synthesized using random hexamer primers and M-MuLV Reverse Transcriptase. Following second-strand cDNA synthesis with DNA Polymerase I and RNase H, the double-stranded cDNA was purified. The purified cDNA underwent end repair, A-tailing, and ligation to Illumina adapters. cDNA fragments of 400–500 bp were selected using AMPure XP beads (Beckman Coulter, USA), followed by PCR amplification and a final purification step with AMPure XP beads to obtain the final sequencing library. Library quality was assessed using an Agilent 2100 Bioanalyzer with the Agilent High Sensitivity DNA Kit. Concentration was initially determined via PicoGreen quantification (Quant-iT PicoGreen dsDNA Assay Kit, Invitrogen) on a Quantifluor-ST fluorometer (Promega), and then precisely quantified by qPCR to obtain the effective library concentration. Finally, the pooled libraries were subjected to paired-end sequencing (PE150) on an Illumina platform.

### Transcriptomic analysis

To ensure data quality, raw reads were processed with Fastp (version 0.22.0) to remove adapter sequences and trim low-quality bases. Reads with an average quality score below Q20 were filtered out, yielding a set of high-quality clean reads for subsequent analysis. Gene function was annotated against multiple databases: NR (NCBI non-redundant protein sequences), GO (Gene Ontology), KEGG (Kyoto Encyclopedia of Genes and Genomes), eggNOG (evolutionary genealogy of genes: Non-supervised Orthologous Groups), Swiss-Prot, Pfam. For expression quantification, clean reads from each sample were aligned to the assembled unigene sequences using RSEM (v2.15). The number of reads mapped to each gene was counted and normalized to calculate FPKM values. Differential expression analysis between comparative groups was performed with DESeq (v1.38.3). Genes with an absolute |log2FoldChange|> 1 and *P*-value < 0.05 were defined as differentially expressed genes (DEGs). Functional enrichment analysis of DEGs was conducted separately. GO term enrichment was performed using top GO (v2.50.0), applying the hypergeometric test to identify significantly over-represented biological functions (*P*-value < 0.05). KEGG pathway enrichment analysis was carried out using the clusterProfiler package (v4.6.0), with pathways meeting a significance threshold of *P*-value < 0.05 considered significantly enriched.

### Metabolomic analysis

Metabolite extraction was performed with slight modifications to a standard protocol. Briefly, each tissue sample (25 mg) was accurately weighed on a pre-cooled balance and transferred to a low-temperature homogenizer. A 500 μL aliquot of extraction solvent (methanol: acetonitrile: water = 2:2:1, v/v). The mixture was homogenized thoroughly and then centrifuged at 12,000 × g for 15 min at 4 °C. The resulting supernatant was collected for subsequent analysis. Chromatographic separation was achieved using a Vanquish UHPLC system (Thermo Fisher Scientific) equipped with a Waters ACQUITY UPLC BEH Amide column (2.1 mm × 50 mm, 1.7 μm). The column temperature was maintained at 40 °C. Mass spectrometric analysis was conducted on an Orbitrap Exploris 120 mass spectrometer (Thermo Fisher Scientific) operating in both positive and negative ionization modes. The instrument was controlled by Xcalibur software (v4.4, Thermo Fisher Scientific), and data were acquired in full MS/DD-MS^2^ mode. Raw data files were converted from the proprietary format to mzXML t using the MSConvert tool (ProteoWizard software package, v3.0.8789). Subsequent data processing, including peak detection, filtering, and alignment, was performed using the XCMS package in R. This generated a final quantitative matrix of metabolite features. Metabolite identification was conducted by matching the acquired MS/MS spectra and retention times against public spectral libraries, including HMDB, MassBank, LipidMaps, mzCloud, and KEGG. The untargeted metabolomic profiling service was provided by Shanghai Personal Biotechnology Co., Ltd.

### Quantitative real-time PCR (qRT-PCR) validation

To confirm the accuracy of the sequencing data, we selected six candidate differentially expressed genes (DEGs) and measured their expression levels using quantitative real-time PCR (qRT-PCR). Primers were designed based on the transcriptome sequencing results using Primer 3.0 software v0.4.0 (Primer-E Ltd., Plymouth, UK), and their sequences are provided in Supplementary Table 13. The β-actin gene was used as an internal reference for normalization. The qRT-PCR assays were performed using the Advantage® RT-PCR Kit (Takara, Japan) and the AceQ® qPCR SYBR® Green Master Mix (Vazyme, Beijing, China). Each sample was analyzed in triplicate. Gene expression levels were calculated using the 2^−ΔΔCt^ method [89], and differences between groups were compared using an independent samples t-test. Additionally, the consistency between all tested genes and the transcriptome sequencing results was assessed.

## Supplementary Information

Supplementary Material 1: Supplementary Table 1. Supplementary Table 1 Summary of rawdata. Supplementary Table 2. Summary of clean data. Supplementary Table 3. Summary of transcript and unigene. Supplementary Table 4. Functional annotation of unigenes. Supplementary Table 5. Differentially expressed genes between TH4F versus TH8F group. Supplementary Table 6. Differentially expressed genes between TH4T versus TH8T group. Supplementary Table 7. The list of enriched KEGG pathways based on differentially expressed genes in TH4F vs TH8F group. Supplementary Table 8. The list of enriched KEGG pathways based on differentially expressed genes in the TH4T versus TH8T comparison. Supplementary Table 9. Differential Metabolites between TH4F versus TH8F group. Supplementary Table 10. Differential metabolites between TH4T versus TH8T group. Supplementary Table 11. The list of enriched KEGG pathways based on differential metabolites in TH4F vs TH8F group. Supplementary Table 12. The list of enriched KEGG pathways based on differential metabolites in TH4T vs TH8T group. Supplementary Table 13. Primer list for quantitative real-time PCR (qRT-PCR).

Supplementary Material 2: Supplementary Fig. 1. Full view of all metabolites. Full view of all metabolites in the TH4F vs TH8F (A) and TH4T vs TH8T metabolomics (B) analysis. Each colour module represents a class of substances. Supplementary Fig. 2. Plot of PCA and OPLS-DA scores for all detected metabolites in TH4F vs TH8F group. PCA scores of positive mode (A) and negative mode (B). OPLS-DA scores of positive mode (C) and negative mode (D). Supplementary Fig. 3. Plot of PCA and OPLS-DA scores for all detected metabolites in TH4T vs TH8T group. PCA scores of positive mode (A) and negative mode (B). OPLS-DA scores of positive mode (C) and negative mode (D). Supplementary Fig. 4. Validation of candidate DEGs by qRT-PCR of different tissues and seasons in *N. taihangnica*. Supplementary Fig. 5 Validation of* PLZF *and* ID4* gene expression in the testicular tissue of *N. taihangnica* by qRT-PCR. Supplementary Fig. 6. Interannual variation in (A) mean temperature, (B) mean minimum temperature, (C) mean maximum temperature, and (D) mean precipitation averaged over March–November for each year from 2010 to 2017. Climate data were derived from the CRU dataset. The red solid lines represent fitted trends based on generalized linear models (GLMs).

## Data Availability

The data sets of sequencing reads have been deposited in the National Center for Biotechnology Information database (NCBI) and can be retrieved under the GEO accession number PRJNA1423436.

## Abbreviations

NAD^+^: Nicotinamide Adenine Dinucleotide
*FOXO3*: Forkhead box O3 gene
mTOR: Mechanistic Target of Rapamycin
FB: Fat body
HPG: Axis hypothalamic-pituitary–gonadal axis
DEGs: Differentially expressed genes
KEGG: Kyoto encyclopedia of genes and genomes
PCA: Principal component analysis
OPLS-DA: Orthogonal partial least squares-discriminant analysis
DEM: Differential metabolite
RNA-seq: RNA sequencing
HDC: Histidine decarboxylase
BMI: Body Mass Index
MASLD: Dysfunction-associated steatotic liver disease
PPARα: Peroxisome Proliferator-Activated Receptor alpha
PPARγ: Peroxisome Proliferator-Activated Receptor gamma
DPYD: Dihydropyrimidine dehydrogenase
HBP: Hexosamine biosynthesis pathway
UDP-GlcNAc: Uridine Diphosphate N-acetylglucosamine
NAM: Nicotinamide
NR: Nicotinamide riboside
NRK: Nicotinamide riboside kinase
NMN: Nicotinamide mononucleotide
TNFα: Tumor Necrosis Factor alpha
SPHK1: Sphingosine Kinase 1
Sph: Sphingosine
S1P: Sphingosine-1-phosphate
PKCε: Protein Kinase C epsilon
GPCR: G Protein-Coupled Receptor
*PLCB*: Phospholipase C beta gene
*KNG1*: Kininogen 1 gene
UDPG: UDP-glucose
*S1P2*: Sphingosine-1-phosphate receptor 2 gene
*S1P4*: Sphingosine-1-phosphate receptor 4 gene
PI3K: Phosphoinositide 3-kinase
MAPK: Mitogen-Activated Protein Kinase
PDK1: 3-Phosphoinositide-Dependent Protein Kinase 1
MEK1: Mitogen-Activated Protein Kinase Kinase 1
mTORC1: MTOR complex 1
mTORC2: MTOR complex 2
PE: Phosphatidylethanolamine
SIRT1: Silent Information Regulator 1
GLM: Generalized linear models
*PLZF*: Promyelocytic leukemia zinc finger gene
*ID4*: Inhibitor of DNA binding 4 gene
SSC: Spermatogonial stem cell
