# Supplementary material for: Multi-omics unveils seasonal remodeling and metabolic crosstalk between testis and abdominal fat body in a non-amplexus stream frog Nanorana taihangnica (Anura: Dicroglossidae)
Source: BMC Genomics. 2026 Jun 22;27:734. doi: 10.1186/s12864-026-13080-4 (PMC13543386; doi:10.1186/s12864-026-13080-4)
Supplement: Supplementary file 1 [file 12864_2026_13080_MOESM1_ESM.zip › Supplementary Table 13.docx]

| **Primer Name** | **Sequence (5'-3')** | **Gene name** |
| --- | --- | --- |
| TRINITY_DN1961_c0_g1-F | AGATTACTGGACTATGAAC | *Mtor* |
| TRINITY_DN1961_c0_g1-R | GAGGATGTGAGGAATAAG |  |
| TRINITY_DN322_c2_g1-F | ACGGTTCTTATCCTTCTTAT | *ENPP3* |
| TRINITY_DN322_c2_g1-R | CGGCTCCTCAATATACAA |  |
| TRINITY_DN9308_c0_g1-F | GACTACTCTCTTCTACAA | *DEPTOR* |
| TRINITY_DN9308_c0_g1-R | ATCAACCAACTAACAATC |  |
| TRINITY_DN1431_c0_g1-F | GTATCTCTGACACTATCG | *Plcb3* |
| TRINITY_DN1431_c0_g1-R | TTCATCTTCCTCATCTTC |  |
| TRINITY_DN4233_c12_g1-F | CATACTCCTAACCGAACATA | *Pdpk1* |
| TRINITY_DN4233_c12_g1-R | CCACACTTCCTGAATCTT |  |
| TRINITY_DN3127_c0_g1-F | ACATTATTCTGAGGAGTGA | *foxo3* |
| TRINITY_DN3127_c0_g1-R | TGGAGCAAGTTCTGATTA |  |
| TRINITY_DN712_c1_g1.p1-F | GGTGATTATGGTGGATACTC | *PLZF* |
| TRINITY_DN712_c1_g1.p1-R | GTGTAATGTTGGCTGCTA |  |
| TRINITY_DN7718_c0_g1.p1 -F | CCACCATTCCGCAGAACA | *ID4* |
| TRINITY_DN7718_c0_g1.p1 -R | GCAGGTCCAGGATGTAGTC |  |
| TRINITY_DN57678_c1_g2-F | ATGTATGTCGTTGGTGTCA | *gapdh* |
| TRINITY_DN57678_c1_g2-R | CAGTTGGTGGTACAGGAG |  |

**Supplementary Table 13 Primer list for quantitative real-time PCR (qRT-PCR)**
