# Supplementary material for: Multi-omics unveils seasonal remodeling and metabolic crosstalk between testis and abdominal fat body in a non-amplexus stream frog Nanorana taihangnica (Anura: Dicroglossidae)
Source: BMC Genomics. 2026 Jun 22;27:734. doi: 10.1186/s12864-026-13080-4 (PMC13543386; doi:10.1186/s12864-026-13080-4)
Supplement: Supplementary file 1 [file 12864_2026_13080_MOESM1_ESM.zip › Supplementary Table 3.docx]

|  | **Transcript** | **Unigene** |
| --- | --- | --- |
| **Total Length (bp)** | 452190165 | 189023260 |
| **Sequence Number** | 405560 | 203177 |
| **Max. Length (bp)** | 22544 | 22544 |
| **Mean Length (bp)** | 1114.98 | 930.34 |
| **N50 (bp)** | 1715 | 1337 |
| **N50 Sequence Number** | 74219 | 36625 |
| **N90 (bp)** | 453 | 395 |
| **N90 Sequence Number** | 282417 | 148125 |
| **GC%** | 44.21 | 43.42 |

**Supplementary Table 3 Summary of transcript and unigene**
