# Supplementary material for: Multi-omics unveils seasonal remodeling and metabolic crosstalk between testis and abdominal fat body in a non-amplexus stream frog Nanorana taihangnica (Anura: Dicroglossidae)
Source: BMC Genomics. 2026 Jun 22;27:734. doi: 10.1186/s12864-026-13080-4 (PMC13543386; doi:10.1186/s12864-026-13080-4)
Supplement: Supplementary file 1 [file 12864_2026_13080_MOESM1_ESM.zip › Supplementary Table 4.docx]

**Supplementary Table 4 Functional annotation of unigenes**

| **Database** | **Number** | **Percentage(%)** |
| --- | --- | --- |
| NR | 55553 | 27.34 |
| GO | 38388 | 18.89 |
| KEGG | 27768 | 13.67 |
| Pfam | 30088 | 14.81 |
| eggNOG | 51744 | 25.47 |
| Swissprot | 39013 | 19.2 |
| In all database | 17996 | 8.86 |
