# Supplementary material for: Multi-omics unveils seasonal remodeling and metabolic crosstalk between testis and abdominal fat body in a non-amplexus stream frog Nanorana taihangnica (Anura: Dicroglossidae)
Source: BMC Genomics. 2026 Jun 22;27:734. doi: 10.1186/s12864-026-13080-4 (PMC13543386; doi:10.1186/s12864-026-13080-4)
Supplement: Supplementary file 1 [file 12864_2026_13080_MOESM1_ESM.zip › Supplementary Table 1.docx]

| **Sample** | **Reads Number** | **Bases (bp)** | **Q30 (bp)** | **N(%)** | **Q20(%)** | **Q30(%)** |
| --- | --- | --- | --- | --- | --- | --- |
| TH4F1 | 39903942 | 6025495242 | 5788669940 | 0.013356 | 98.48 | 96.07 |
| TH4F2 | 37870344 | 5718421944 | 5487802875 | 0.013293 | 98.45 | 95.97 |
| TH8F1 | 58273306 | 8799269206 | 8497732780 | 0.013377 | 98.75 | 96.57 |
| TH8F2 | 51005940 | 7701896940 | 7443585343 | 0.013371 | 98.78 | 96.65 |
| TH4T1 | 37156046 | 5610562946 | 5380943895 | 0.013363 | 98.42 | 95.91 |
| TH4T2 | 39808578 | 6011095278 | 5770822461 | 0.013324 | 98.46 | 96.00 |
| TH8T1 | 46765720 | 7061623720 | 6802015838 | 0.013375 | 98.62 | 96.32 |
| TH8T2 | 63742990 | 9625191490 | 9279237150 | 0.013429 | 98.64 | 96.41 |

**Supplementary Table 1 Summary of raw Data**
