# Supplementary material for: Multi-omics unveils seasonal remodeling and metabolic crosstalk between testis and abdominal fat body in a non-amplexus stream frog Nanorana taihangnica (Anura: Dicroglossidae)
Source: BMC Genomics. 2026 Jun 22;27:734. doi: 10.1186/s12864-026-13080-4 (PMC13543386; doi:10.1186/s12864-026-13080-4)
Supplement: Supplementary file 1 [file 12864_2026_13080_MOESM1_ESM.zip › Supplementary Table 2.docx]

**Supplementary Table 2 Summary of clean Data**

| **Sample** | **Clean Reads Number** | **Clean Data (bp)** | **Clean Reads(%)** | **Clean Data(%)** | **Clean GC number** | **Clean GC content(%)** |
| --- | --- | --- | --- | --- | --- | --- |
| TH4F1 | 39107062 | 5885156973 | 98.00 | 97.67 | 2624315374 | 44.60 |
| TH4F2 | 37097494 | 5582618666 | 97.96 | 97.63 | 2491438293 | 44.63 |
| TH8F1 | 57399314 | 8639938147 | 98.50 | 98.19 | 4005674270 | 46.37 |
| TH8F2 | 50247214 | 7566156319 | 98.51 | 98.24 | 3510139467 | 46.40 |
| TH4T1 | 36361842 | 5470186027 | 97.86 | 97.50 | 2442623423 | 44.66 |
| TH4T2 | 39007248 | 5867359159 | 97.99 | 97.61 | 2619207953 | 44.65 |
| TH8T1 | 45959442 | 6912750798 | 98.28 | 97.89 | 3170030311 | 45.86 |
| TH8T2 | 62583856 | 9422437562 | 98.18 | 97.89 | 4315060558 | 45.80 |
