# Supplementary material for: Multi-omics unveils seasonal remodeling and metabolic crosstalk between testis and abdominal fat body in a non-amplexus stream frog Nanorana taihangnica (Anura: Dicroglossidae)
Source: BMC Genomics. 2026 Jun 22;27:734. doi: 10.1186/s12864-026-13080-4 (PMC13543386; doi:10.1186/s12864-026-13080-4)
Supplement: Supplementary file 2 [file 12864_2026_13080_MOESM2_ESM.zip › Supplementary Figure 4.pdf]

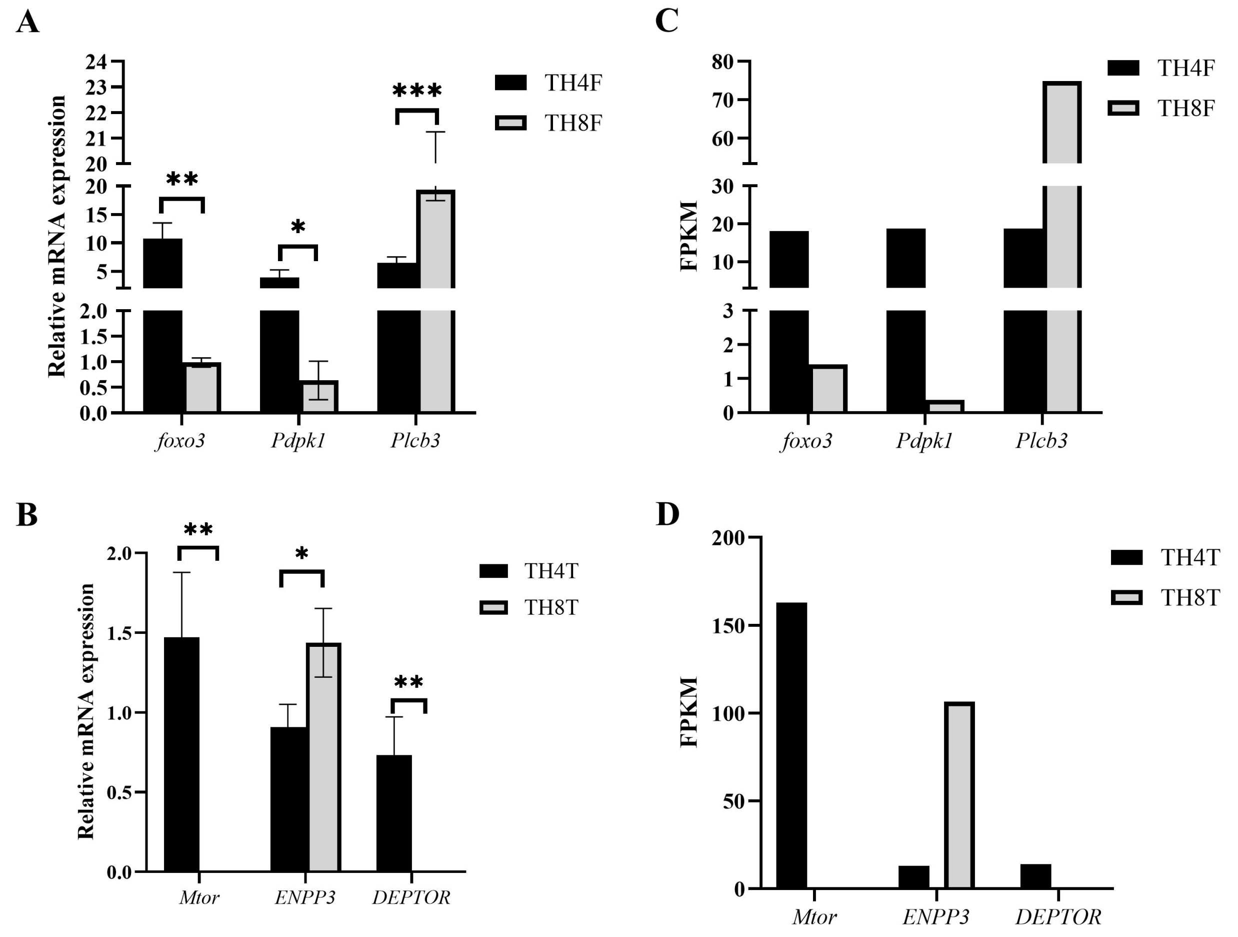

A
Relative mRNA expression
TH4F
TH8F
**
*
***
foxo3
Pdpk1
Plcb3
C
FPKM
TH4F
TH8F
foxo3
Pdpk1
Plcb3
B
Relative mRNA expression
TH4T
TH8T
**
*
**
Mtor
ENPP3
DEPTOR
D
FPKM
TH4T
TH8T
Mtor
ENPP3
DEPTOR
