# Supplementary material for: Multi-omics unveils seasonal remodeling and metabolic crosstalk between testis and abdominal fat body in a non-amplexus stream frog Nanorana taihangnica (Anura: Dicroglossidae)
Source: BMC Genomics. 2026 Jun 22;27:734. doi: 10.1186/s12864-026-13080-4 (PMC13543386; doi:10.1186/s12864-026-13080-4)
Supplement: Supplementary file 2 [file 12864_2026_13080_MOESM2_ESM.zip › Supplementary Fig. 5.pdf]

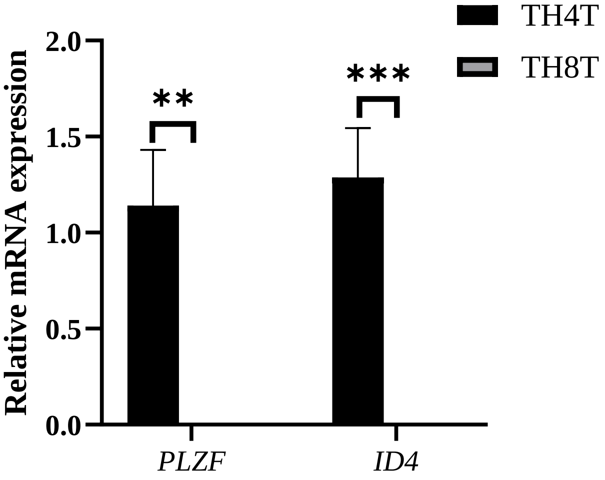

TH4T
TH8T
Relative mRNA expression
2.0
1.5
1.0
0.5
0.0
**
***
PLZF
ID4
