# Supplementary material for: Multi-omics unveils seasonal remodeling and metabolic crosstalk between testis and abdominal fat body in a non-amplexus stream frog Nanorana taihangnica (Anura: Dicroglossidae)
Source: BMC Genomics. 2026 Jun 22;27:734. doi: 10.1186/s12864-026-13080-4 (PMC13543386; doi:10.1186/s12864-026-13080-4)
Supplement: Supplementary file 2 [file 12864_2026_13080_MOESM2_ESM.zip › Supplementary Fig. 6.pdf]

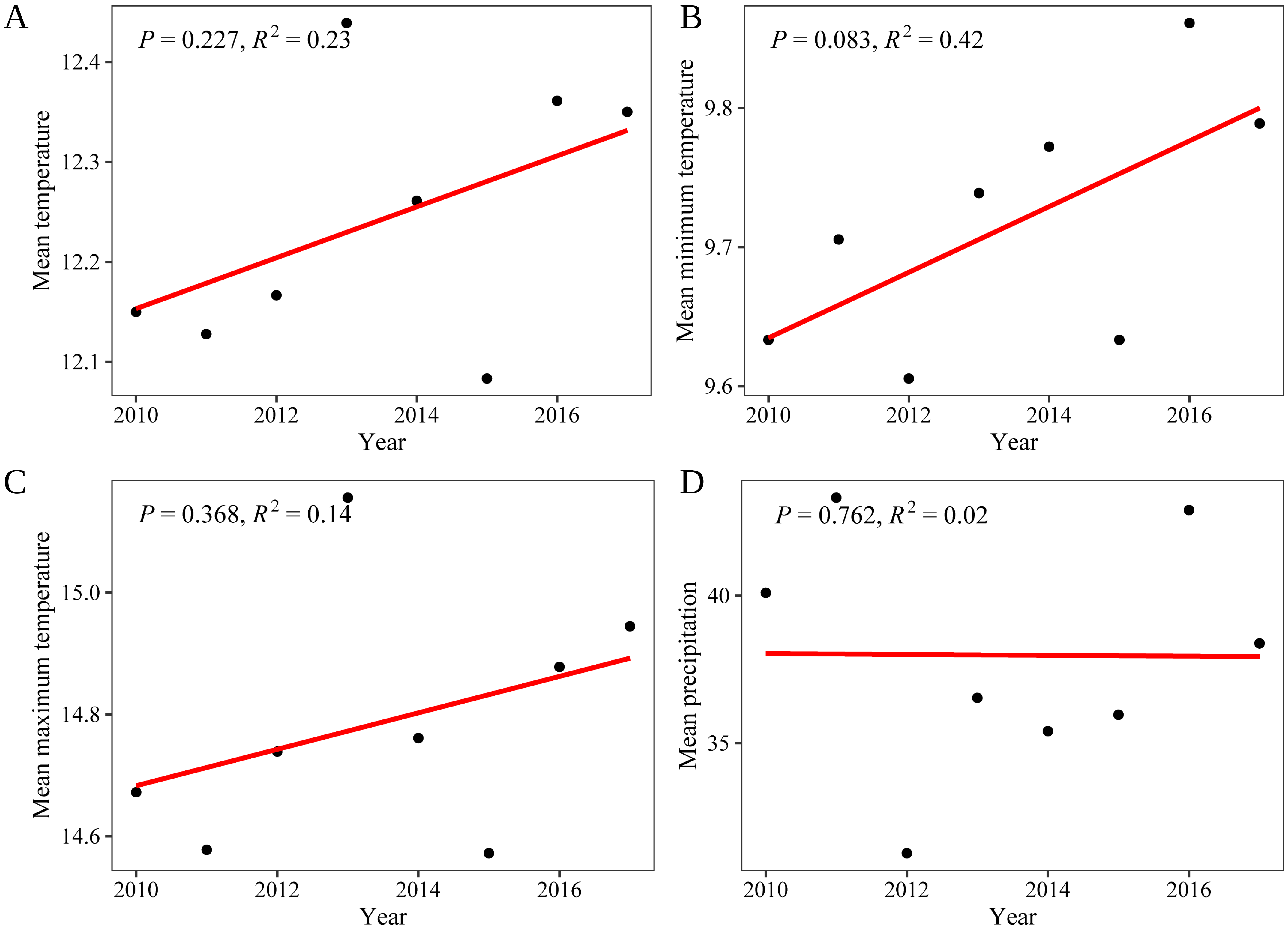

A
$P = 0.227$, $R^2 = 0.23$
Mean temperature
12.4
12.3
12.2
12.1
2010
2012
2014
2016
Year
B
$P = 0.083$, $R^2 = 0.42$
Mean minimum temperature
9.8
9.7
9.6
2010
2012
2014
2016
Year
C
$P = 0.368$, $R^2 = 0.14$
Mean maximum temperature
15.0
14.8
14.6
2010
2012
2014
2016
Year
D
$P = 0.762$, $R^2 = 0.02$
Mean precipitation
40
35
2010
2012
2014
2016
Year
