# Supplementary material for: Multi-omics unveils seasonal remodeling and metabolic crosstalk between testis and abdominal fat body in a non-amplexus stream frog Nanorana taihangnica (Anura: Dicroglossidae)
Source: BMC Genomics. 2026 Jun 22;27:734. doi: 10.1186/s12864-026-13080-4 (PMC13543386; doi:10.1186/s12864-026-13080-4)
Supplement: Supplementary file 2 [file 12864_2026_13080_MOESM2_ESM.zip › Supplementary Figure 1.pdf]

A

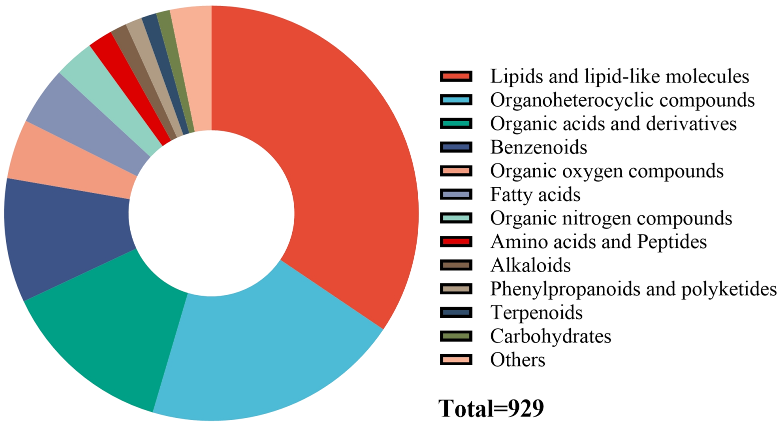
Lipids and lipid-like molecules
Organoheterocyclic compounds
Organic acids and derivatives
Benzenoids
Organic oxygen compounds
Fatty acids
Organic nitrogen compounds
Amino acids and Peptides
Alkaloids
Phenylpropanoids and polyketides
Terpenoids
Carbohydrates
Others
Total=929

B

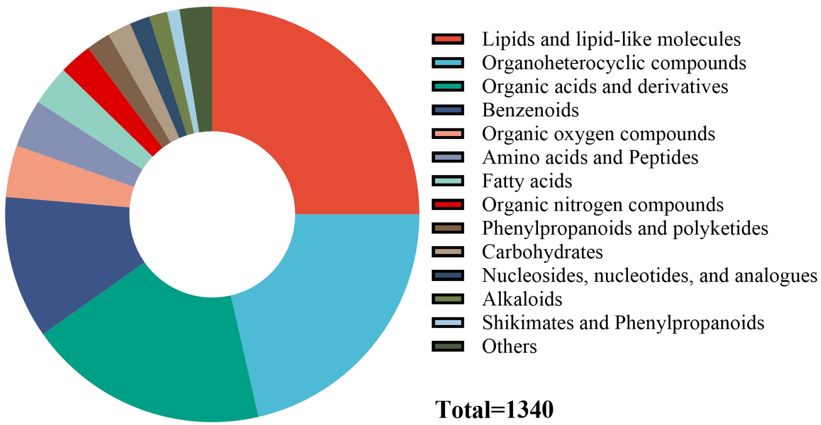
Lipids and lipid-like molecules
Organoheterocyclic compounds
Organic acids and derivatives
Benzenoids
Organic oxygen compounds
Amino acids and Peptides
Fatty acids
Organic nitrogen compounds
Phenylpropanoids and polyketides
Carbohydrates
Nucleosides, nucleotides, and analogues
Alkaloids
Shikimates and Phenylpropanoids
Others
Total=1340
