# Supplementary material for: Multi-omics unveils seasonal remodeling and metabolic crosstalk between testis and abdominal fat body in a non-amplexus stream frog Nanorana taihangnica (Anura: Dicroglossidae)
Source: BMC Genomics. 2026 Jun 22;27:734. doi: 10.1186/s12864-026-13080-4 (PMC13543386; doi:10.1186/s12864-026-13080-4)
Supplement: Supplementary file 2 [file 12864_2026_13080_MOESM2_ESM.zip › Supplementary Figure 2.pdf]

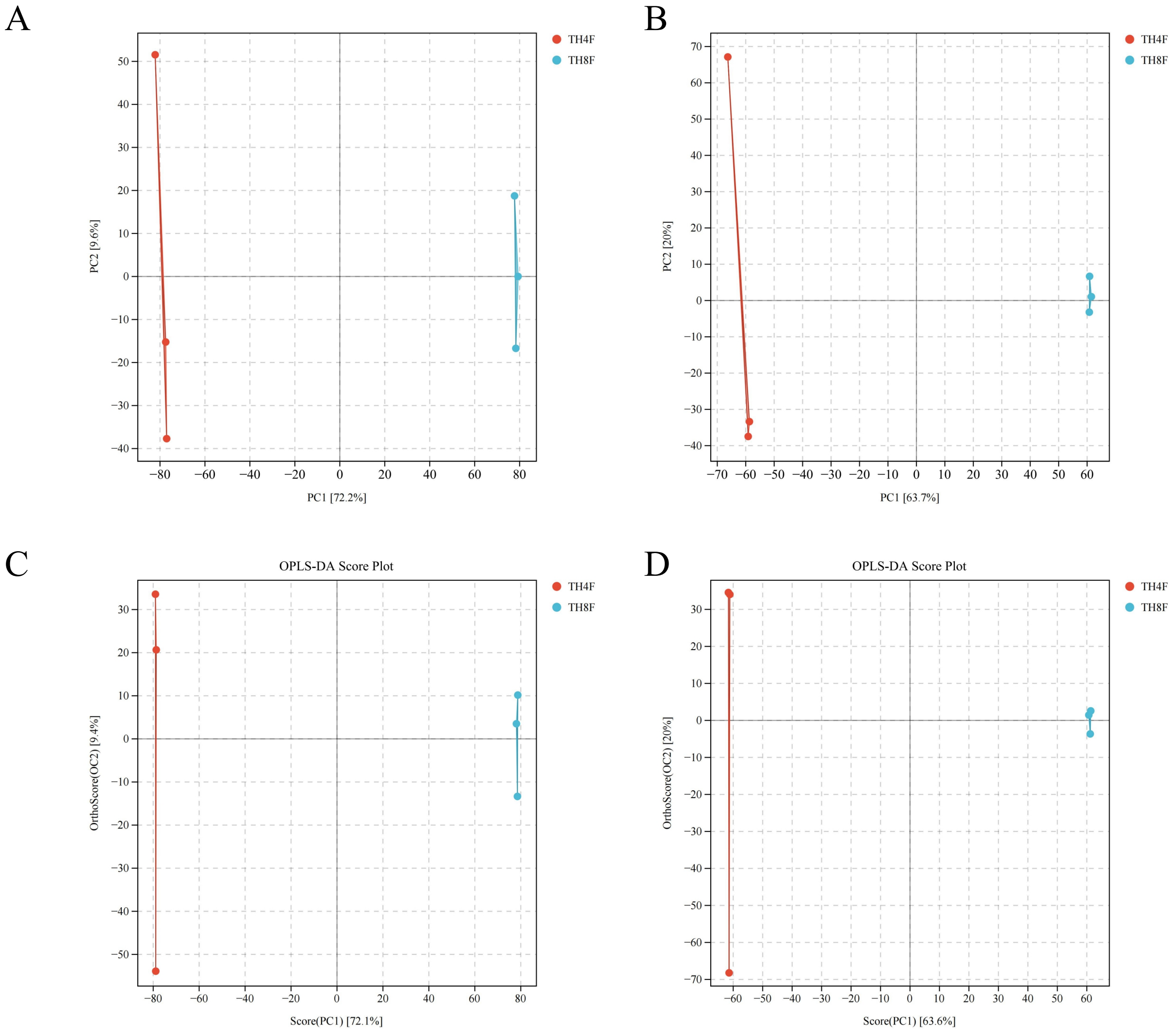
A
TH4F
TH8F
PC2 [9.6%]
PC1 [72.2%]
B
TH4F
TH8F
PC2 [20%]
PC1 [63.7%]
C
OPLS-DA Score Plot
TH4F
TH8F
OrthoScore(OC2) [9.4%]
Score(PC1) [72.1%]
D
OPLS-DA Score Plot
TH4F
TH8F
OrthoScore(OC2) [20%]
Score(PC1) [63.6%]
