# Supplementary material for: Multi-omics unveils seasonal remodeling and metabolic crosstalk between testis and abdominal fat body in a non-amplexus stream frog Nanorana taihangnica (Anura: Dicroglossidae)
Source: BMC Genomics. 2026 Jun 22;27:734. doi: 10.1186/s12864-026-13080-4 (PMC13543386; doi:10.1186/s12864-026-13080-4)
Supplement: Supplementary file 2 [file 12864_2026_13080_MOESM2_ESM.zip › Supplementary Figure 3.pdf]

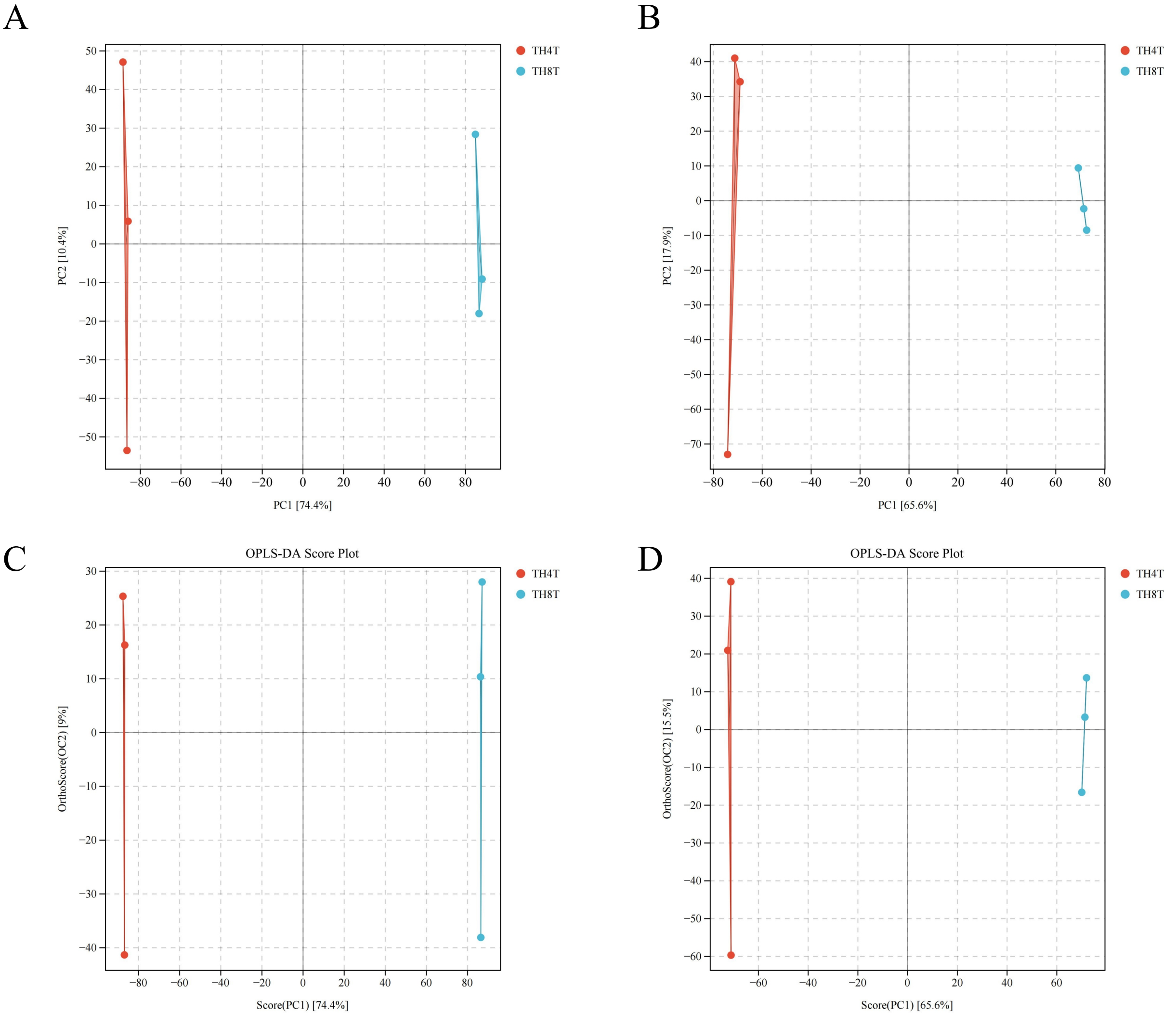
A
B
C
D
TH4T
TH8T
PC1 [74.4%]
PC2 [10.4%]
PC1 [65.6%]
PC2 [17.9%]
OPLS-DA Score Plot
Score(PC1) [74.4%]
OrthoScore(OC2) [9%]
Score(PC1) [65.6%]
OrthoScore(OC2) [15.5%]
